# Structural basis for PtdInsP_2_-mediated human TRPML1 regulation

**DOI:** 10.1038/s41467-018-06493-7

**Published:** 2018-10-10

**Authors:** Michael Fine, Philip Schmiege, Xiaochun Li

**Affiliations:** 1 Department of Physiology, University of Texas Southwestern Medical Center, Dallas, TX 75390 USA; 2 Department of Molecular Genetics, University of Texas Southwestern Medical Center, Dallas, TX 75390 USA; 3 Department of Biophysics, University of Texas Southwestern Medical Center, Dallas, TX 75390 USA

## Abstract

Transient receptor potential mucolipin 1 (TRPML1), a lysosomal channel, maintains the low pH and calcium levels for lysosomal function. Several small molecules modulate TRPML1 activity. ML-SA1, a synthetic agonist, binds to the pore region and phosphatidylinositol-3,5-bisphosphate (PtdIns(3,5)P_2_), a natural lipid, stimulates channel activity to a lesser extent than ML-SA1; moreover, PtdIns(4,5)P_2_, another natural lipid, prevents TRPML1-mediated calcium release. Notably, PtdIns(3,5)P_2_ and ML-SA1 cooperate further increasing calcium efflux. Here we report the structures of human TRPML1 at pH 5.0 with PtdIns(3,5)P_2_, PtdIns(4,5)P_2_, or ML-SA1 and PtdIns(3,5)P_2_, revealing a unique lipid-binding site. PtdIns(3,5)P_2_ and PtdIns(4,5)P_2_ bind to the extended helices of S1, S2, and S3. The phosphate group of PtdIns(3,5)P_2_ induces Y355 to form a π-cation interaction with R403, moving the S4–S5 linker, thus allosterically activating the channel. Our structures and electrophysiological characterizations reveal an allosteric site and provide molecular insight into how lipids regulate TRP channels.

## Abstract (web summary)

Transient receptor potential mucolipin 1 (TRPML1) is a lysosomal channel which maintains the low pH and calcium levels for lysosomal function. Here authors use structural biology and electrophysiology to show how lipids bind and allosterically activate TRPML1.

## Introduction

TRP channels, which include six sub-families, are widely regulated by various sensations and ligands^1^. Of these ligands, the TRP family has demonstrated regulation by more than 50 endogenous lipids^2^. TRPML1 regulates lysosomal calcium signaling, lipid trafficking, and autophagy-related processes^3–5^. Loss-of-function TRPML1 mutants cause a neurodegenerative disorder, Mucolipidosis type IV (MLIV)^6–8^. While recent structural determinations provide insight into the regulatory mechanisms of TRPs by small molecules^9–12^, there is still limited structural detail showing how specific lipids regulate activity. Determination of the structural regulation of TRP channels by endogenous lipid ligands can provide a wealth of insight into the physiological function of these channels^13^.

In previous studies, PtdIns(3,5)P_2_, a low-abundance phosphoinositol in late endosomes and lysosomes^14^, stimulates the opening of TRPML1, specifically at low pH^12,15,16^. In contrast, PtdIns(4,5)P_2_, a similarly structured phosphoinositol lipid abundant in the plasma membrane, decreases the TRPML1-mediated calcium release on the cell surface^16^. Notably, human fibroblasts derived from patients with Niemann-Pick C disease (a lysosomal storage disease caused by dysfunctional lipid trafficking), exhibit decreased TRPML1 activity^15^, implying a correlation between lysosomal abnormality and TRPML1 behaviors. Therefore, structural insights into TRPML1 regulation by lipid ligands represent a strong opportunity to study the lipid-mediated regulatory mechanism among TRP channels.

**Fig. 1:**
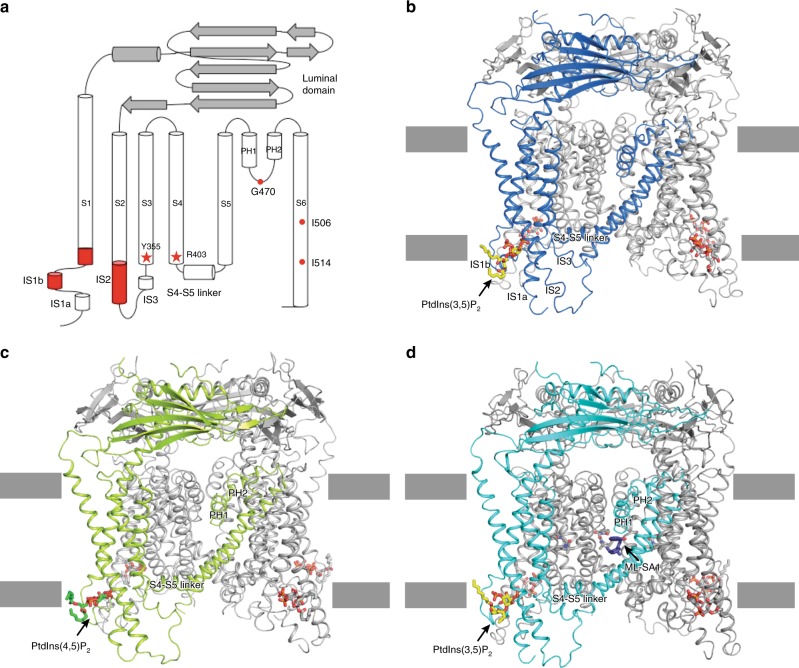
The overall structures of hTRPML1 with different ligands. **a** The secondary structure of hTRPML1. The major structural elements are labeled and the key residues for the PtdInsP_2_ activation or in the pore regions are indicated. **b** The structure of hTRPML1 with PtdIns(3,5)P_2_ (yellow sticks). **c** The structure of hTRPML1 with PtdIns(4,5)P_2_ (green sticks). **d** The structure of hTRPML1 with PtdIns(3,5)P_2_ (yellow sticks) and ML-SA1 (blue sticks)

Recently, several groups reported structures of mammalian TRPML1 and TRPML3^11,12,17–20^. TRPML1 forms a classic tetramer: each subunit contains six transmembrane helices (S1–S6), two pore helices (PH1 and PH2) and a ~30 kD lumenal/extracellular domain (Fig. 1a). The extensions of S1–S3 (labeled as IS1–IS3), including several basic amino acids, present an unique feature that presumably binds to PtdIns(3,5)P_2_ in the membrane^16–18^. However, there is no atomic detail for the interaction between the PtdInsP_2_ and TRPMLs.

In this manuscript, we report three structures of human TRPML1 with (1) PtdIns(3,5)P_2_, (2) PtdIns(4,5)P_2_, and (3) ML-SA1 with PtdIns(3,5)P_2_ at 3.5–3.7-Å resolution, revealing a lipid-binding site to allosterically activate the channel, distinct from the previously reported site in TRPV1^9,21^. These structures along with electrophysiological characterizations reveal an allosteric site and illuminate how lipids regulate TRP channels.

## Results

### Assembly of TRPML1 with distinct ligands for cryo-EM study

To investigate the role of inositides in TRPML1 activation, we first assembled the human TRPML1 protein in nanodiscs containing PtdIns(3,5)P_2_ and used cryo-EM to detect the conformation of TRPML1 with PtdIns(3,5)P_2_. Unfortunately, the particles failed to reconstitute a structure at atomic resolution. To address this, we purified the protein in a low pH digitonin buffer and independently incubated with either PtdIns(3,5)P_2_, PtdIns(4,5)P_2_, or PtdIns(3,5)P_2_ plus ML-SA1 to capture TRPML1 in its various ligand-bound states. The resulting three structures (PtdIns(3,5)P_2_, PtdIns(4,5)P_2_, and PtdIns(3,5)P_2_ with ML-SA1; Fig. 1b–d and Supplementary Table 1) revealed a distinct inositide binding pocket for TRPML1 and yielded structural insight into one possible mechanism of channel regulation.

### Structure of PtdIns(3,5)P_2_-bound TRPML1

**Fig. 2:**
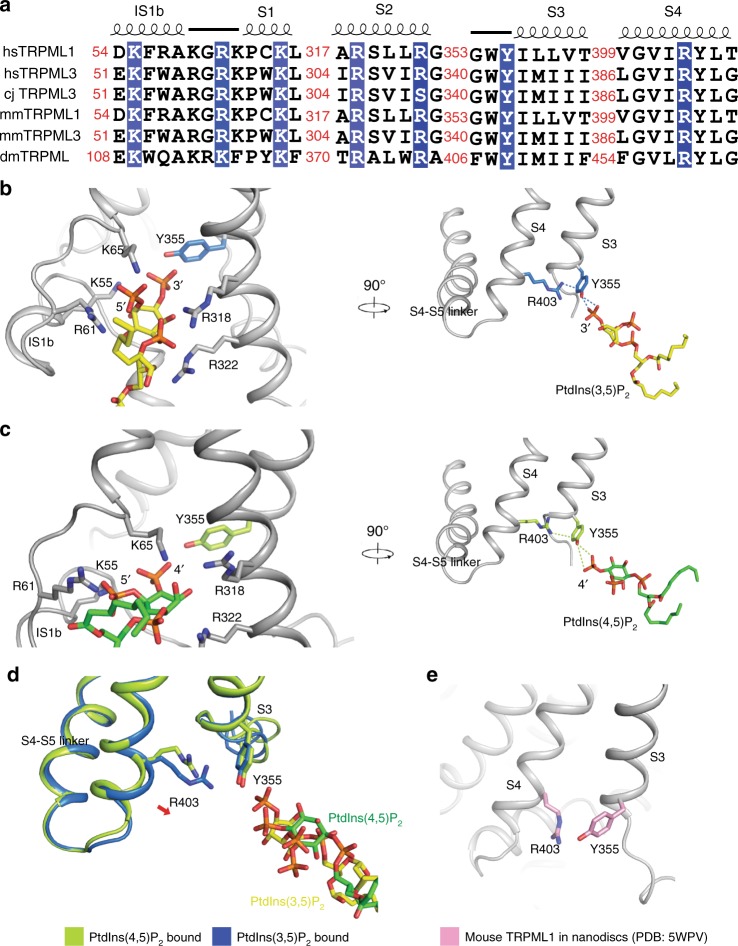
The molecular details of hTRPML1 bound to PtdInsP_2_. **a** The sequence alignment of the key residues for PtdInsP_2_ recognition among multiple TRPMLs. **b** The interaction details of hTRPML1 bound to PtdIns(3,5)P_2_. **c** The interaction details of hTRPML1 bound to PtdIns(4,5)P_2_. **d** The structural comparison of hTRPML1 with PtdIns(3,5)P_2_ or PtdIns(4,5)P_2_. **e** Interaction details of R403 and Y355 (pink) in mouse TRPML1 closed conformation in nanodiscs (PDB: 5WPV)

The cryo-EM structure of PtdIns(3,5)P_2_ bound TRPML1 is detected in a closed conformation at 3.5 Å resolution (Supplementary Fig. 1). An electron density is observed in the cavity created by extensions of S1, S2, and S3 (Supplementary Fig. 2). Our previously reported ML-SA1 bound structure in the absence of PtdIns(3,5)P_2_ lacked an extra density in this area at a similar resolution, leading us to putatively identify this density as PtdIns(3,5)P_2_^12^. Single channel recordings of mouse TRPML1 activity indicated that the open probability of TRPML1 remained quite low when the PtdIns(3,5)P_2_ was bound, with less than half of the channel population presented in an open conformation^18^. This finding suggests that it may be difficult to capture significant populations of PtdIns(3,5)P_2_-mediated opening of TRPML1 for cryo-EM structural determinations. K55, R61, and K65 in S1, and R318 and R322 in S2 are involved in PtdIns(3,5)P_2_ binding (Fig. 2a). Mutations on these corresponding residues in TRPML3^17^ and the deletion of IS1 and IS2 in mouse TRPML1^18^ reduces the response of TRPMLs to PtdIns(3,5)P_2_. In addition to the residues in S1 and S2, the side chain density of Y355 in the PtdIns(3,5)P_2_-bound structure is adjacent to the density of the phosphate group of PtdIns(3,5)P_2_, implying a strong binding between these two groups (Fig. 2b and Supplementary Fig. 2). This interaction enriches the negative change of the aromatic ring plane of Y355, which recruits the head of R403 in S4 forming a π-cation bond (Fig. 2b). As R403 locates in the C-terminus of S4, it might induce a shift to move the S4–S5 linker away from the pore center.

### Structure of PtdIns(4,5)P_2_-bound TRPML1

In order to compare the binding sites of PtdIns(3,5)P_2_ and PtdIns(4,5)P_2_, we determined the structure of PtdIns(4,5)P_2_ bound TRPML1 at 3.6 Å resolution (Supplementary Figs. 3, 4). Supporting our proposed inositide binding site, PtdIns(4,5)P_2_ binds to the same area of TRPML1 as PtdIns(3,5)P_2_ (Fig. 2c). However, the 3′ phosphate group of PtdIns(3,5)P_2_ is closer to Y355 than the 4′ phosphate group in the PtdIns(4,5)P_2_-bound structure (Fig. 2d). This difference may cause the charge of the tyrosine ring in the PtdIns(4,5)P_2_-bound structure to be less negative than in the PtdIns(3,5)P_2_-bound structure, preventing the strong π-cation interaction with R403 (Fig. 2d). Y355 and R403 in the structure of TRPML1 in nanodiscs do not form a π-cation interaction in the absence of PtdIns(3,5)P_2_^18^ (Fig. 2e). From these observations we hypothesize that one potential structural mechanism behind the differential regulation of TRPML1 by PtdIns(4,5)P_2_ and PtdIns(3,5)P_2_ may lie in the 3′ phosphate supporting the formation of a π-cation interaction between Y355 and R403. Previous studies showed that PtdIns(4,5)P_2_ can decrease the ion efflux of TRPML1 by competing with PtdIns(3,5)P_2_ binding to the channel^16,18^. This is consistent with our structural observation that PtdIns(3,5)P_2_ and PtdIns(4,5)P_2_ bind the same area. PtdIns(4,5)P_2_ may serve as an agonist blocker on the cell surface, preventing the other lipid agonist from entering the lipid-binding site, therefore keeping the channel inactive. In normal cells, PtdIns(3,5)P_2_ and PtdIns(4,5)P_2_ are not abundant on the same membrane, and the activity of TRPML1 cation efflux depends on its cellular location.

### Validation of PtdIns(3,5)P_2_-mediated TRPML1 activation

**Fig. 3:**
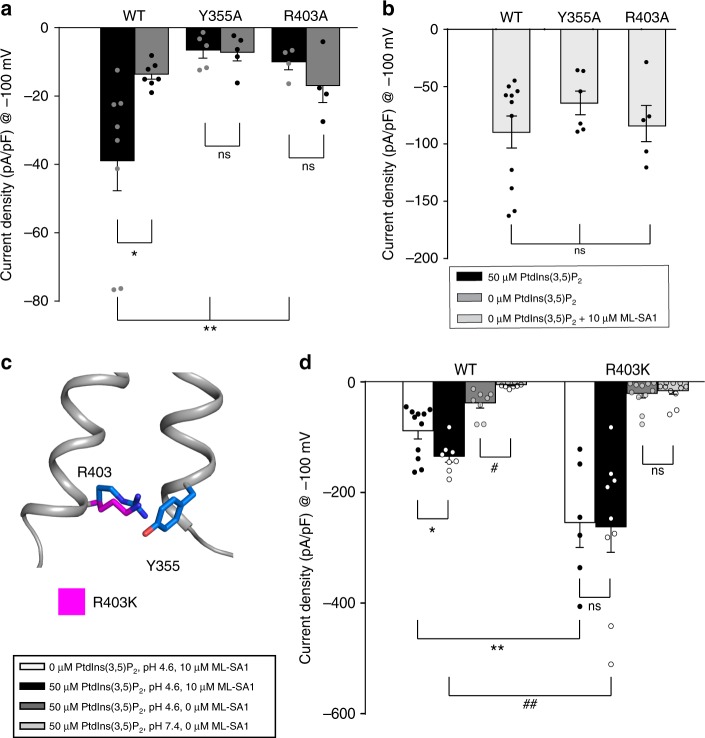
TRPML1 channel activation for WT-L/A and Inositide binding mutants. **a** Current densities at −100 mV for various TRPML1 constructs with or without cytoplasmic PtdIns(3,5)P_2_ reveal significant stimulation for WT but not the inositide pore binding mutants Y355A and R403A when 50 µM PtdIns(3,5)P_2_ is present in the pipette solution. (*p = 0.038; **p < 0.005); (*n* = 8, 7, 5, 5, 4, 4, resp). **b** Stimulation with the synthetic agonist ML-SA1 (10 µM); (*n* = 11, 6, 5 resp). **c** Structural view of the R403 and Y355 residues (blue) and the R403K mutant (magenta). **d** Current densities at −100 mV for various WT-L/A and R403K TRPML1 mutant with or without 50 µM PtdIns(3,5)P_2_ or 10 µM ML-SA1 at pH 4.6 and 7.4. The R403K mutation leads to a significant increase in current densities when ML-SA1 is applied regardless of the presence of inositide yet does not significantly increase channel current densities when ML-SA1 is not present. (*p = 0.023; #*p* = 0.001; **p < 0.001; ##*p* = 0.008); (*n* = 11, 8, 8, 6, 9, 6, 11, 11, resp). Values are mean ± s.e.m

To test our observation and hypothesis, we generated two eGPF-fused TRPML1-L/A^22^ mutations, TRPML1-Y355A-LA and TRPML1-R403A-L/A. The four leucine-to-alanine mutations in TRPML1-L/A have been reported to increase cell surface localization for subsequent electrophysiological characterization via whole-cell patch clamp^22^. With PtdIns(3,5)P_2_ perfused in the cytoplasmic solution, there is a significant increase in current density for HEK cells expressing WT-L/A at low pH (Fig. 3a). However, both the Y355A-L/A and R403A-L/A mutants show no significant increase in channel activity at low pH when 50 µM PtdIns(3,5)P_2_ is present in the pipette (Fig. 3a).

Earlier reports describe a significant reduction of PtdIns(3,5)P_2_ mediated TRPML1 current at higher pH in the presence of 1 mM Ca^2+^^20^. Among all the channels tested, only WT-L/A channels demonstrated this significant reduction in current density at pH 7.4 (Supplementary Figure 5a) further supporting our observation of PtdIns(3,5)P_2_ mediated TRPML1 function. To verify channel function for all WT-L/A and inositide binding mutant channels, application of the synthetic agonist ML-SA1 was used to open the channel in the absence of perfused PtdIns(3,5)P_2_ (Fig. 3b and Supplementary Fig. 5b). All expressed constructs were stimulated by 10 μM ML-SA1 with current densities 2–3 times higher than for WT-LA channels with PtdIns(3,5)P_2_ application alone, similar to our previously reported findings^12^ and providing validation of proper channel surface expression and function. This data coupled with our structural observations and previous work on TRPML3 and PtdIns(3,5)P_2_^17^, suggest an alternate site for inositide binding separate from ML-SA1 that can allosterically affect channel opening. Notably, since an R403 mutation causes MLIV, it is likely that this mutant cannot be activated by PtdIns(3,5)P_2_.

Previous studies showed that PtdIns(3,5)P_2_ and ML-SA1 cooperate to stimulate TRPML1 considerably more than either agonist alone^12,15^. To investigate whether or not the π-cation interaction is involved in this potentiation, we utilized the ability of lysine to form a stronger π-cation interaction than the endogenous arginine^23^. Compared with arginine, lysine has a shorter side chain that presents less flexibility and an amine group with a more compressed positive charge, theoretically making it easier to capture the aromatic ring of Y355 and form a stronger π-cation interaction (Fig. 3c).

We expressed this mutant and compared the current with WT channels with and without PtdIns(3,5)P_2_ or ML-SA1. In the presence of 10 μM ML-SA1, current densities of both WT and R403K were greatly enhanced (Fig. 3d and Supplementary Fig. 5a). Cytoplasmic supplementation of PtdIns(3,5)P2 significantly potentiates WT current, but not R403K current. Regardless of the presence or absence of cytoplasmic PtdIns(3,5)P2, the R403K stimulation with ML-SA1 is significantly higher than WT. Interestingly, in the absence of ML-SA1, the R403K mutant was not significantly stimulated by PtdIns(3,5)P_2_ at pH 4.6, unlike WT channels (Fig. 3d and Supplementary Fig. 5a). This leads us to speculate whether the PtdIns(3,5)P2 interaction may induce additional confirmations not directly supported by R403 and Y355 interactions. This could help explain why the open probability of the PtdIns(3,5)P_2_ stimulated channel remains low as well as the difficulty in detecting an open conformation with only inositide binding^18^. We hypothesize that the gain of function mutation R403K does not necessarily support inositide signaling in the absence of ML-SA1. Instead the mutation may enhance the π-cation interaction supporting robust stimulation when ML-SA1 is applied regardless of PtdIns(3,5)P_2_.

### Structure of PtdIns(3,5)P_2_/ML-SA-1-bound TRPML1

**Fig. 4:**
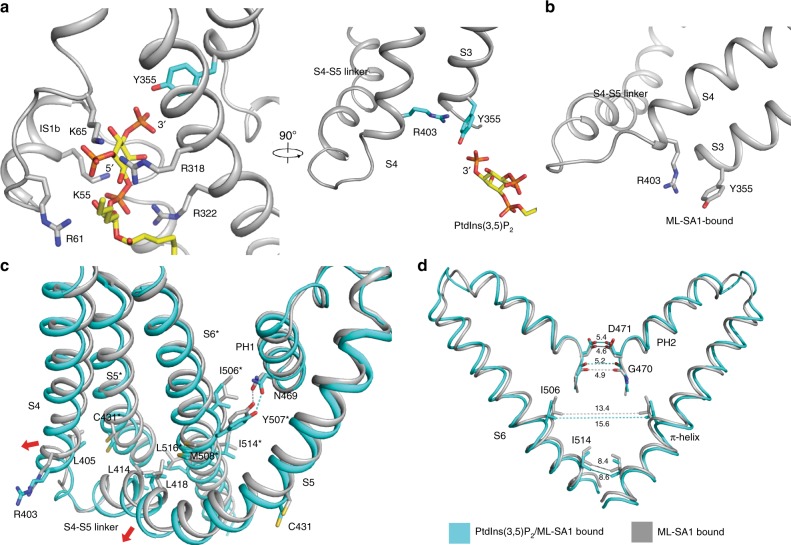
The putative mechanism of PtdIns(3,5)P_2_ and ML-SA1 cooperation. **a** PtdIns(3,5)P_2_ induces the π-cation interaction of Y355 and R403 in PtdIns(3,5)P_2_/ML-SA1 bound structure. **b** The molecular detail of Y355 and R403 in the ML-SA1 bound structure (PDB: 5WJ9). **c** Structural comparison of both agonists bound (cyan) and the ML-SA1 bound (gray) hTRMPL1 structures. **d** The comparison of the pore region of both agonists bound and the ML-SA1 bound (gray) hTRMPL1 structures

To capture the mechanism of ML-SA1 and PtdIns(3,5)P_2_ cooperation, the ML-SA1/PtdIns(3,5)P_2_-bound structure was determined at 3.7 Å resolution in an open conformation (Supplementary Figs. 6, 7). In this structure Y355 binds the 3′ phosphate group of PtdIns(3,5)P_2_ (Fig. 4a) and S1–S3 have a conformational shift due to PtdIns(3,5)P_2_ binding when compared to the ML-SA1 alone structure (Supplementary Fig. 8a). The comparison between the PtdIns(3,5)P_2_ /ML-SA1 and PtdIns(3,5)P_2_-bound structures shows that ML-SA1 binding causes the S3 and S4 of the PtdIns(3,5)P_2_ binding site to move away from the pore center (Supplementary Fig. 8b). In the ML-SA1-bound structure, the ring of Y355 does not align with the polar head of R403; consequently, there is no π-cation interaction between Y355 and R403 (Fig. 4b). Although, the density of the R403 side chain is not clear in the density map (Supplementary Fig. 7), structural comparison still suggests that the shift of R403 may trigger a movement of the S4–S5 linker and channel pore, since the S4–S5 linker moves 2–3 Å away from the pore (Fig. 4c). When PtdIns(3,5)P_2_ binds TRPML1, R403 induces a shift of L405, L414, and L418, which have hydrophobic interactions with C431 in the S5, and L516 and M508 in the S6 of neighboring subunit, allosterically forcing the S5 and S6 away from the pore center (Fig. 4c). The movement of L516 can facilitate the opening of I514 in the lower gate; the shift of M508 causes the π-helix opening by I506; and Y507 can form a hydrophilic interaction with N469, opening the selectivity filter (Fig. 4d). Although PtdIns(3,5)P_2_ cannot directly bind to the S4–S5 linker, where the agonists of TRPV1 bind and allosterically affect the opening of TRPV1^21^, the allosteric activation mechanism of TRPV1 and TRPML1 still present similar behavior: the movement of the S4–S5 affects the opening of the channel pore. Interestingly, in mouse TRPML1-nanodisc structures, this linker presents two distinct conformations, presumably reflecting a different allosteric regulation of the S4–S5 linker in the lipid environment^18^.

## Discussion

In this manuscript, we report three human TRPML1 structures with different ligands. TRPML1 employs its extensions of IS1–IS3 to recognize the PtdInsP_2_ binders using basic amino acids. PtdIns(3,5)P_2_ induces Y355 in S3 to form a π-cation interaction with R403 in S4 through the binding of its 3′ phosphate group to the hydroxyl group of Y355. This π-cation interaction affects the movement of the S4–S5 linker, a common allosteric site among TRP channels. Compared with ML-SA1, which directly forces the pore open, this stimulation is indirect and mild, explaining the low proportion of TRPML1 channels that are active with PtdIns(3,5)P_2_. The fact that the R403K mutant appears to facilitate ML-SA1 pore opening but does not significantly affect PtdInsP_2_ mediated channel opening poses an interesting hypothesis. It is possible that the inositide binding site allosterically regulates channel opening in several different ways. As noted by earlier reports, the idea that both PtdInsP_2_ bind to the same pocket but have significantly different effects on channel opening could represent a way to control channel activity through localization within different lipid enriched compartments. In this manner endogenous TRPML1 is kept relatively inactive at the surface of the cell due to an abundance of PtdIns(4,5)P_2_. As TRPML1 is trafficked to the lysosome, the membrane is enriched with PtdIns(3,5)P_2_. Concurrently, PtdIns(4,5)P_2_ is displaced by PtdIns(3,5)P_2_ allowing the formation of the observed π-cation interaction. In this conformation, the channel is more susceptible to pore opening by agonists like ML-SA1. However, this does not rule out the possibility of additional open conformations supported by PtdIns(3,5)P_2_ binding_._ As the R403K mutation does not increase channel activity, it is also possible these conformations may not rely on the π-cation formed between R403 and Y355.

The structures of other TRP channels have revealed several lipid-binding sites among the different channels. The structure of TRPML3 in the closed conformation with amphipols shows several sterol-like molecules attached near the pore region including our observed ML-SA1-binding pocket (Supplementary Fig. 9a). However, there is no lipid density in the extensions of IS1–IS3^17^. The structure of TRPV6 in the open conformation reveals a lipid bound to the S4–S5 linker, the allosteric site of the channel, presumably stimulating channel opening^10^ (Supplementary Fig. 9b). Similar to TRPV6, the structure of TRPV1 in nanodiscs reveals an associated lipid bound to the pore region consistent with the ML-SA1-binding pocket of TRPML1 and a phosphatidylinositol lipid was observed in the S4–S5 linker of TRPV1 although it is also in a closed conformation^9^ (Supplementary Fig. 9c). Recently, the structure of TRPV5 with PtdIns(4,5)P_2_ in an open conformation has been reported revealing a unique binding site between the N-linker, S4–S5 linker and S6 helix of TRPV5^24^ (PDB: 6DMU). In our reported structures, there is no lipid density in the S4–S5 linker implying this site may not be used for the channel activation among TRPML family. Instead of the S4–S5 linker, the allosteric site for TRPML activation resides in the extensions of IS1–IS3, which capture the PtdInsP_2_ lipid.

Previous reports showed that other ion channels, such as GIRK2^25^, Kir2.2^26^, and TPC1^27^, are also regulated by PtdInsP_2_. Compared with those structures, our structures reveal a binding site far away from either the pore or the S4–S5 linker that can open the channel remotely. It is possible that TRPML1 is evolutionarily suitable for binding PtdIns(3,5)P_2_ due to the high abundance of PtdIns(3,5)P_2_ in the lysosomal environment. Thus, our structural observations show an allosteric site among TRP channels that can host lipid ligands, and may reveal one molecular mechanism for lipid-mediated TRPMLs regulation.

## Methods

### Protein expression and purification

Human TRPML1 was cloned into pEG BacMam with an N-terminal Flag tag. The protein was expressed using baculovirus-mediated transduction of mammalian HEK293S GnTI^−^ cells (ATCC). These cells tested negative for mycoplasma contamination. At 48 h post infection at 37 °C, cells were disrupted by sonication in buffer A (20 mM HEPES, pH 7.0, 150 mM NaCl) with 1 mM PMSF and 5 μg ml^−1^ each of leupeptin and aprotinin. After low-speed centrifugation at 3470 g, the resulting supernatant was incubated in buffer A with 1% (w/v) lauryl maltose neopentyl glycol (MNG, Anatrace) for 1 h at 4 °C. The lysate was centrifuged at 34,572 g and the supernatant was loaded onto an anti-Flag M2 affinity column (Sigma). After washing twice, the protein was eluted in 20 mM HEPES, pH 7.0, 150 mM NaCl, 0.1 mg ml^−1^ Flag peptide and 0.01% MNG, and then concentrated. The protein was purified by Superdex-200 size-exclusion chromatography (GE Healthcare) in a buffer containing 20 mM sodium acetate, pH 5.0, 150 mM NaCl and 0.06% (w/v) digitonin (Sigma). The peak fractions were collected and concentrated to 5–7 mg/ml for grid preparation. The mutated DNA constructs were generated using QuikChange Mutagenesis Kit (Agilent) (Supplementary Table 2).

### Whole-cell patch clamp electrophysiology

The full-length human TRPML1 (L/A) was subcloned to pEGFP-C1 for electrophysiological assays. Whole-cell patch clamp recordings of cell electrical parameters were performed using Matlab based Capmeter v7.2^28,29^ with a National Instruments digital acquisition board and an Axopatch 200B patch clamp amplifier. For current density comparisons, capacitance was monitored in real-time using square-wave voltage perturbations (20 mV; 0.5 kHz). Input resistances for the capillary pipettes were 2–10 MΩ, and the apparent cell resistances were 0.5–2 GΩ. External solutions were adjusted to RT (23–25 °C) in gravity-fed parallel solution lines with an outlet flow velocities of 2–5 mm/s allowing extracellular solution changes within 2–3 s. For TPRML1 current–voltage analysis, a voltage ramp was generated from −150 mV to 20 mV over 1.5 s. Voltage ramps were aligned and statistical analysis was performed at −100 mV. Boroscilicate glass pipettes were fire polished and back-filled with the cytoplasmic solutions containing in mM: 120 cesium methanesulfonate, 4 NaCl, 10 EGTA, 2 MgCl_2_, 20 HEPES, pH 7.2 with CsOH (~25 mM). A 5 mM stock solution of C8: PtdIns(3,5)P_2_ (Echelon) was prepared in ddH_2_O and aliquots were stored at −80 °C. For PtdIns(3,5)P_2_ stimulated recordings, a 1:100 dilution of lipid was added to the cytoplasmic solution and briefly sonicated. Extracellular solutions were set to pH 4.6 and contained in mM: 140 sodium gluconate, 5 KCl, 10 glucose, 10 HEPES, 10 MES, 1 MgCl_2_, 1 CaCl_2_, and 8 HCl. For control experiments at pH 7.4, bath solution contained in mM: 140 NaCl, 5 KCl, 10 glucose, 20 HEPES, 1 MgCl_2_, 1 CaCl_2_, and 8 NaOH. A total of 10 µM ML-SA1 was added to the appropriate bath solution immediately prior to recording. Low passage HEK293T (ATCC CRL-11268) cells used for electrophysiology were maintained in DMEM (GIBCO) with 10% FBS, penicillin-streptomycin, and l-glutamine (Sigma), and routinely monitored for mycoplasma infection using MycoSensor PCR kits (Agilent). HEK293T cells were transiently transfected with Lipofectamine 3000 (Invitrogen) and used within 48–72 h. The cells were deplated with trypsin (0.25%) and placed in a bath solution on an inverted Nikon TE2000U inverted microscope equipped with a 60X oil immersion, 1.45-NA objective. A Lambda DG-4 xenon epifluorescence power supply with Semrock FITC filters were used to detect cells expressing surface TRPML1-GFP mutants. Statistical analyses were performed in Matlab and SigmaPlot (SigmaStat) using either a Rank Sum Test or a Student's *t*-test as determined by the SigmaPlot Normality Test. Figures were created in GraphPad Prism 7.05. Current–voltage relations represent the mean of at least 4 voltage ramps, and agonist-induced current density changes represent the mean and s.e.m. of at least four independent cells per condition.

### EM sample preparation and imaging

A protein sample was added to Quantifoil R1.2/1.3 400 mesh Au holey carbon grids (Quantifoil), blotted with Vitrobot Mark IV (FEI), and frozen in liquid ethane. For PtdIns(3,5)P_2_ or PtdIns(4,5)P_2_ bound protein, the protein in a buffer containing 20 mM sodium acetate, pH 5.0, 150 mM NaCl and 0.06% digitonin was incubated with 0.2 mM PtdIns(3,5)P_2_ or PtdIns(4,5)P_2_ (Echelon, dissolved in H_2_O as a 10 mM stock) on ice for 1 h before grid preparation and freezing. For PtdIns(3,5)P_2_/ML-SA1 bound protein, the protein in a buffer containing 20 mM sodium acetate, pH 5.0, 150 mM NaCl and 0.06% digitonin was incubated with 0.2 mM PtdIns(3,5)P_2_ and 0.3 mM ML-SA1 (Tocris Bioscience, dissolved in DMSO as a 20 mM stock) on ice for 1 h before grid preparation and freezing. The grids were imaged with a 300 keV Titan Krios (FEI) with a Gatan K2 Summit direct electron detector (Gatan). The data were collected at 1.07 Å per pixel with a dose rate of eight electrons per physical pixel per second. Images were recorded for 12-s exposure in 30 subframes to give a total dose of 84 electrons per Å^2^.

### Imaging processing and 3D reconstruction

Dark subtracted images were first normalized by gain reference that resulted in a pixel size of 1.07 Å per pixel. Drift correction was performed using the program Unblur^30^. The contrast transfer function (CTF) was estimated using CTFFIND4^31^. After automatic picking and manual micrograph inspection, the particles were extracted for subsequent 2D and 3D classification. Motion correction of individual particles was performed using the program alignparts_lmbfgs^32^. Using the structure of human TRPML1 (EMD-8840) low-pass filtered to 60 Å as the initial model, 3D classification was carried out in RELION^33^. The best classes of four structures, containing 50k~60k particles, provided a 5–6 Å map. Refinement was performed in FREALIGN^34^ using this best class as the initial model and all particles post motion correction to generate the final map for model construction.

### Model construction

To obtain better side-chain densities for model building, we sharpened one half-map using BFACTOR.EXE (author: Nikolaus Grigorieff) with a resolution limit of 3.53 Å (PtdIns(3,5)P_2_ bound) and 3.57 Å (PtdIns(4,5)P_2_ bound) and 3.7 Å (ML-SA1/PtdIns(3,5)P_2_ bound), and a B-factor value of −100 Å^2^. To precisely determine the location of PtdInsP_2_, we took advantage of the stronger signal phosphate atoms have in the electron density compared to other atoms (oxygen, nitrogen, and carbon), and we increased signal to 8–10*σ* level to determine its head position. The entire model was built in COOT^35^.

### Model refinement and validation

The model was refined in real space using PHENIX^36^ and also in reciprocal space using Refmac^37,38^. Structural factors were calculated from a half-map (working) using the program SFall^38^. Fourier shell correlations (FSCs) were calculated between the two half maps, the model against the half1 map, the half2 map, and full map^40^. Local resolutions were estimated using Blocres^41^. MolProbity^42^ was used to validate the geometries of the model. Structure figures were generated using PyMOL (http://www.pymol.org) and Chimera^43^.

## Electronic supplementary material

Supplementary Information

## Data Availability

The data supporting the findings of this manuscript are available from the corresponding author upon reasonable request. The 3D cryo-EM density maps of TRPML1 have been deposited in the Electron Microscopy Data Bank under the accession numbers EMDB-9000 (PI(3,5)P_2_-bound), EMDB-9001 (PI(4,5)P_2_-bound) and EMDB-9002 (PI(3,5)P_2_/ML-SA1-bound). Atomic coordinates for the atomic model of TRPML1 have been deposited in the Protein Data Bank under the accession numbers 6E7P (PI(3,5)P_2_-bound), 6E7Y (PI(4,5)P_2_-bound) and 6E7Z (PI(3,5)P_2_/ML-SA1-bound).
