# Supplementary material for: Structural basis for PtdInsP2-mediated human TRPML1 regulation
Source: Nat Commun. 2018 Oct 10;9:4192. doi: 10.1038/s41467-018-06493-7 (PMC6180102; doi:10.1038/s41467-018-06493-7)
Supplement: Supplementary file 1 — Supplementary Information [file 41467_2018_6493_MOESM1_ESM.pdf]

Supplementary Information

Structural basis for PtdInsP_2-mediated human TRPML1 regulation

Michael Fine, Philip Schmiege, and Xiaochun Li

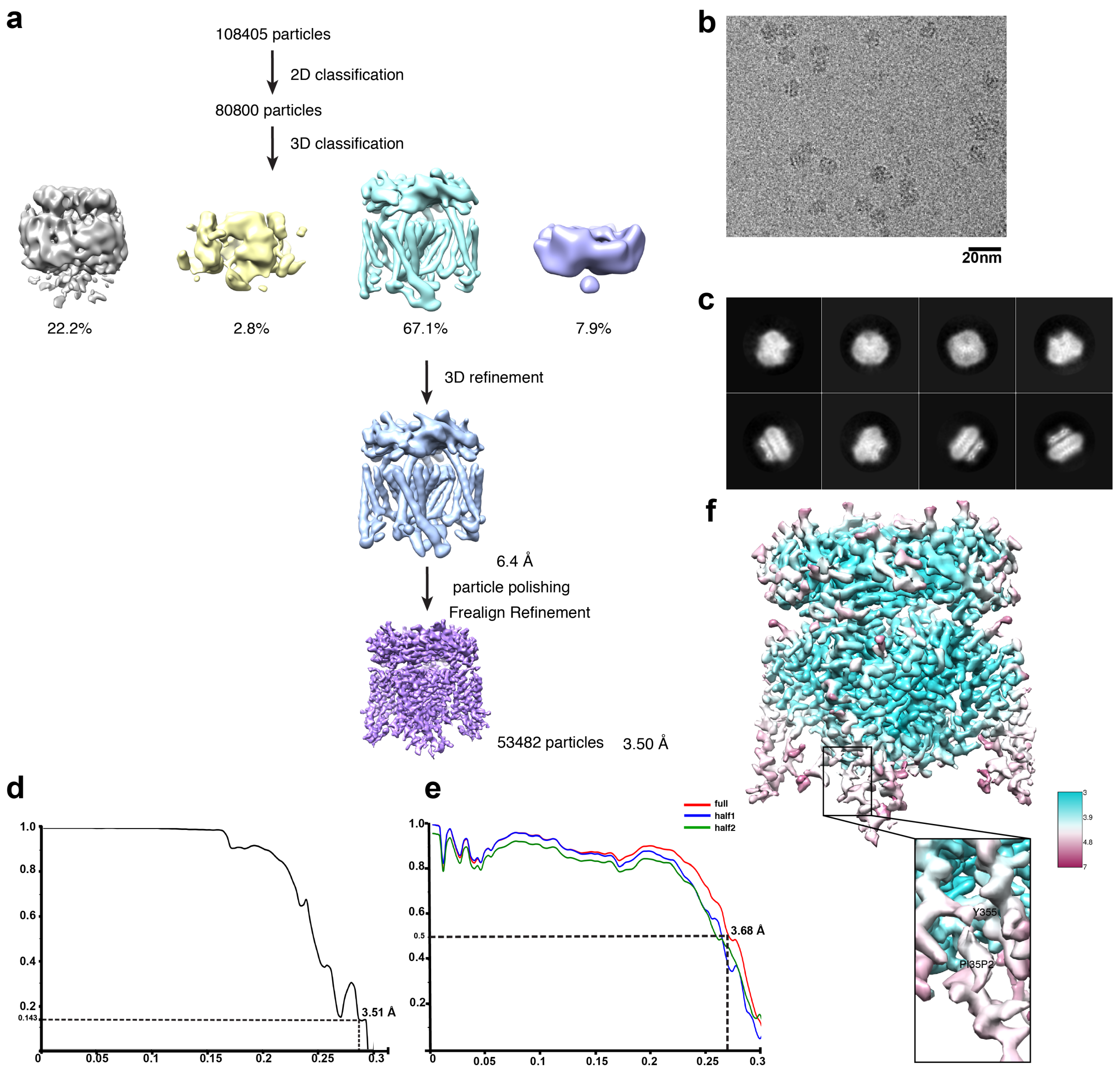

Supplementary Fig. 1 Data processing and model quality assessment of PtdIns(3,5)P_2 bound TRPML1.

a, The data processing work-flow for PtdIns(3,5)P_2 bound TRPML1. **b**, A representative electron micrograph at defocus -2.0 μm. **c,** 2D classification. **d,** Fourier shell correlation (FSC) curve of the structure with FSC as a function of resolution using Frealign output. **e,** The FSC curves calculated between the refined structure and the half map used for refinement, the other half map, and the full map. **f**, Density map of PtdIns(3,5)P_2 bound TRPML1 structure colored by local resolution estimation using blocres.

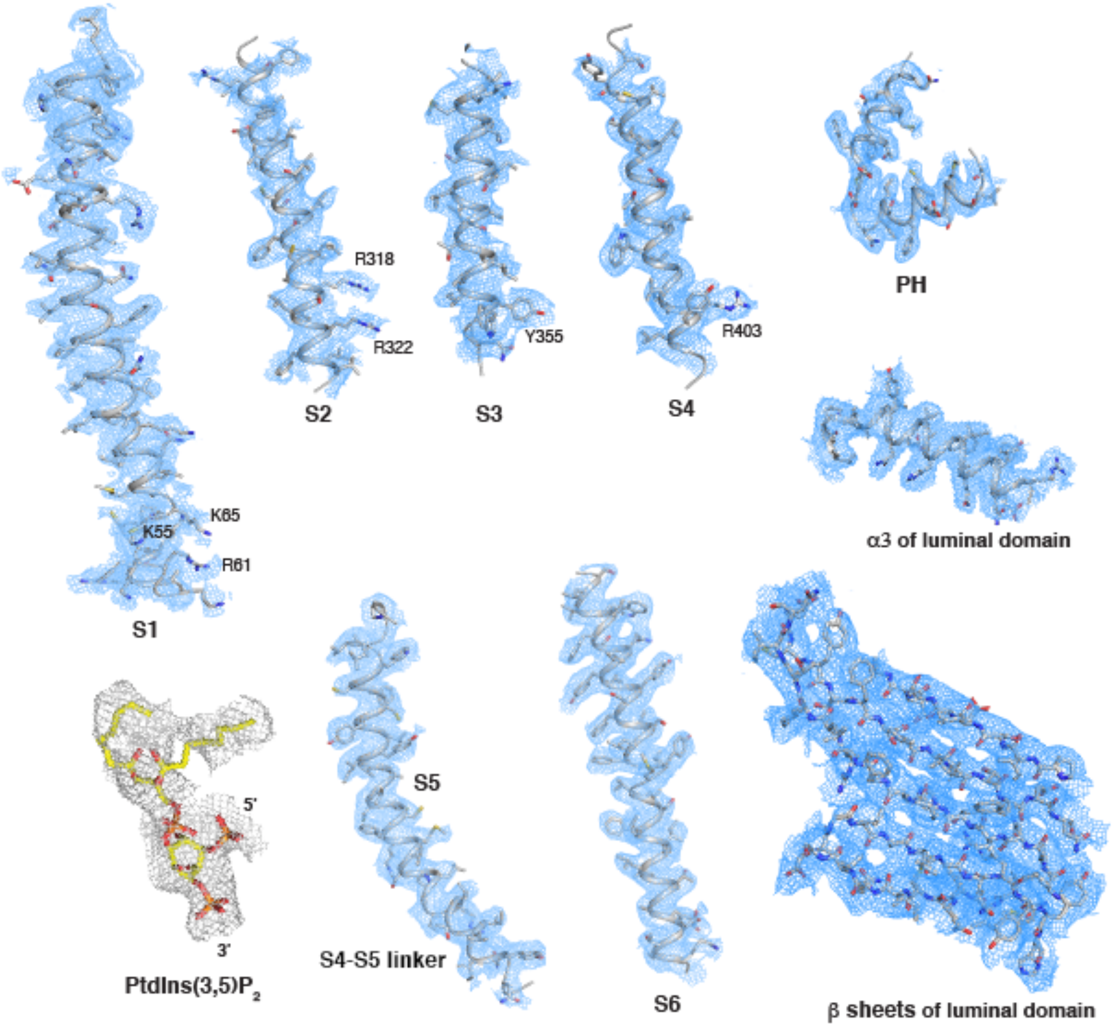

Supplementary Fig. 2 Electron microscopy density of different portions of PtdIns(3,5)P_2 bound TRPML1 at 5σ level.

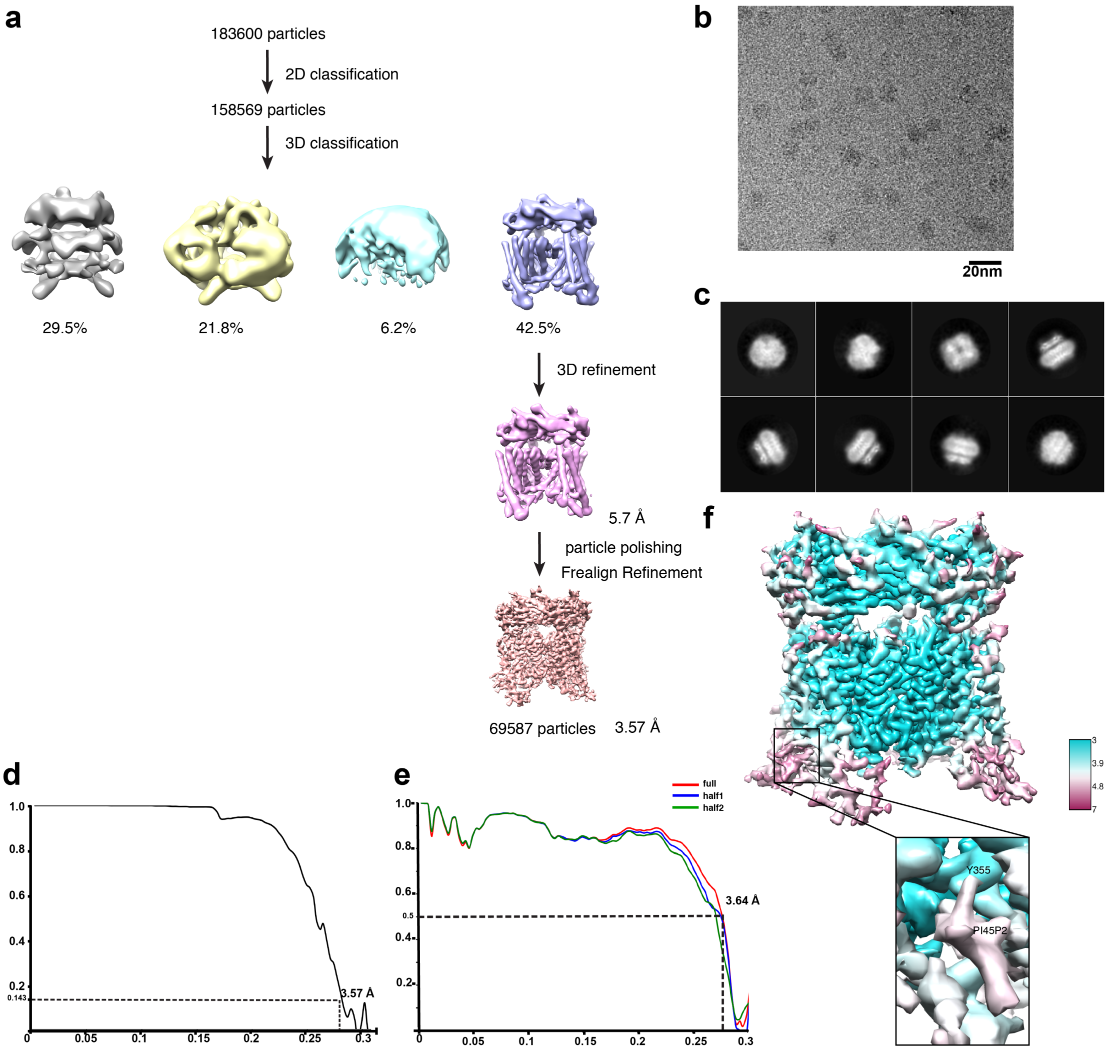

Supplementary Fig. 3 Data processing and model quality assessment of PtdIns(4,5)P_2 bound TRPML1.

a, The data processing work-flow for PtdIns(4,5)P_2 bound TRPML1. **b**, A representative electron micrograph at defocus -2.0 µm. **c,** 2D classification. **d,** Fourier shell correlation (FSC) curve of the structure with FSC as a function of resolution using Frealign output. **e,** The FSC curves calculated between the refined structure and the half map used for refinement, the other half map, and the full map. **f**, Density map of PtdIns(4,5)P_2 bound TRPML1 structure colored by local resolution estimation using blocres.

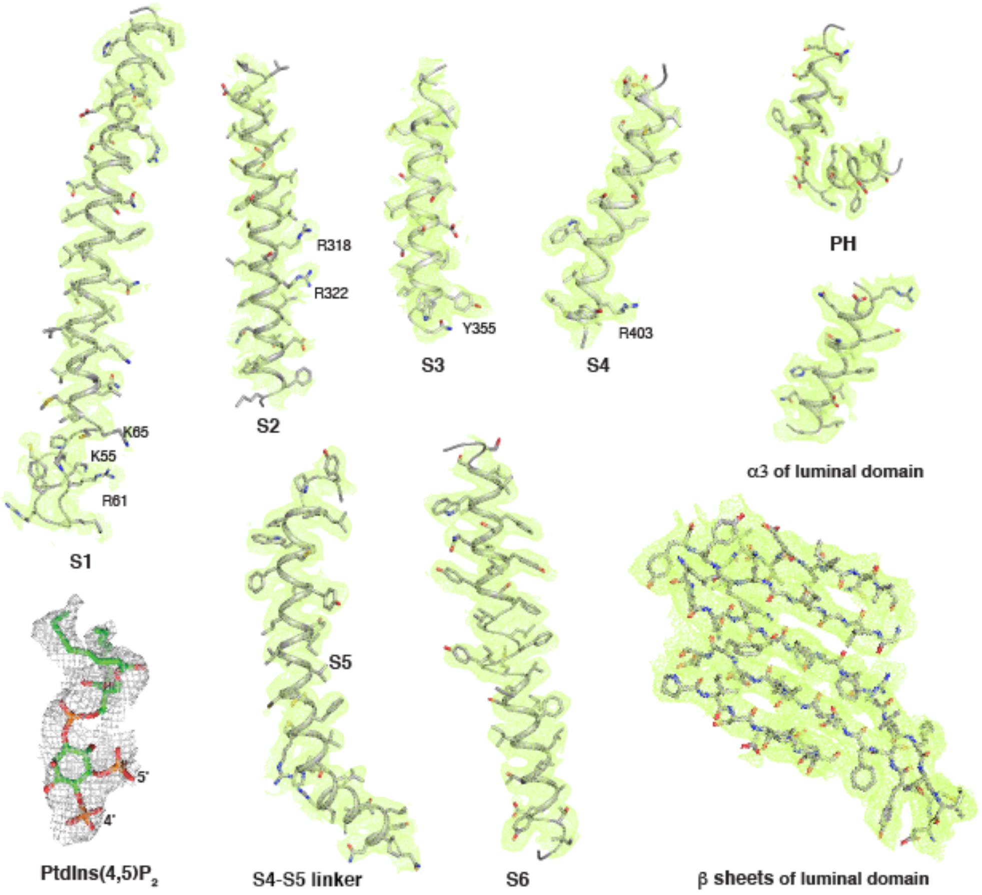

Supplementary Fig. 4 Electron microscopy density of different portions of PtdIns(4,5)P_2 bound TRPML1 at 5σ level.

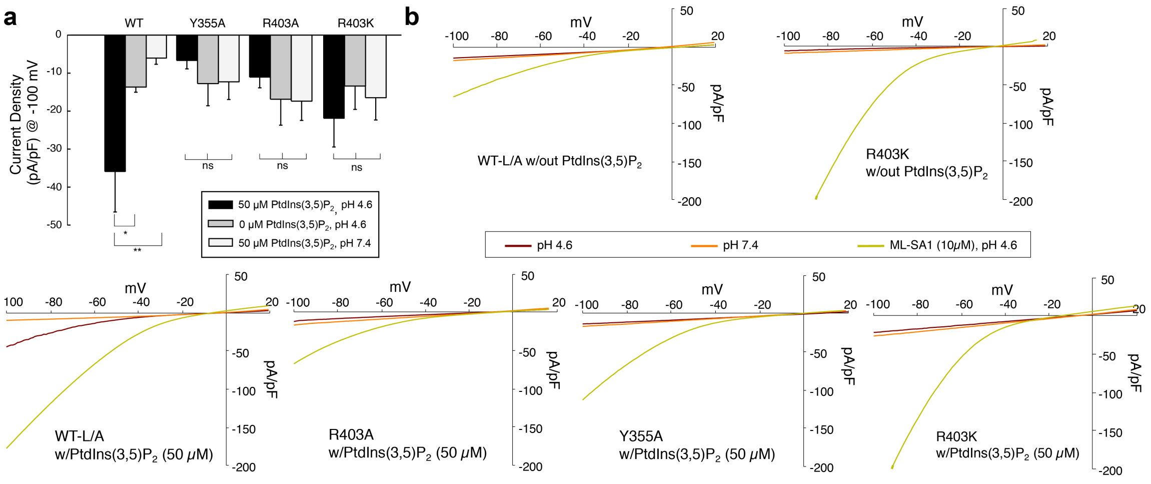

Supplementary Fig. 5 Validation and representative current-voltage relationships of TRPML1 inositide binding mutants

a, Current densities at -100 mV for various TRPML1 constructs with or without 50 µM PtdIns(3,5)P2 at pH 4.6 and 7.4 reveal significant stimulation for WT at low pH as previously published but not the inositide pore binding mutants Y355A, R403A and R403K when 50 µM PtdIns(3,5)P2 is present in the pipette solution. (p*=0.038, p**=0.001); (n=8, 7, 8, 5, 5, 5, 4, 4, 4, 11, 6, 11, resp.). Values are mean ± s.e.m. **b,** Representative current voltage relationships of TRPML1 WT-L/A and inositide binding mutants.

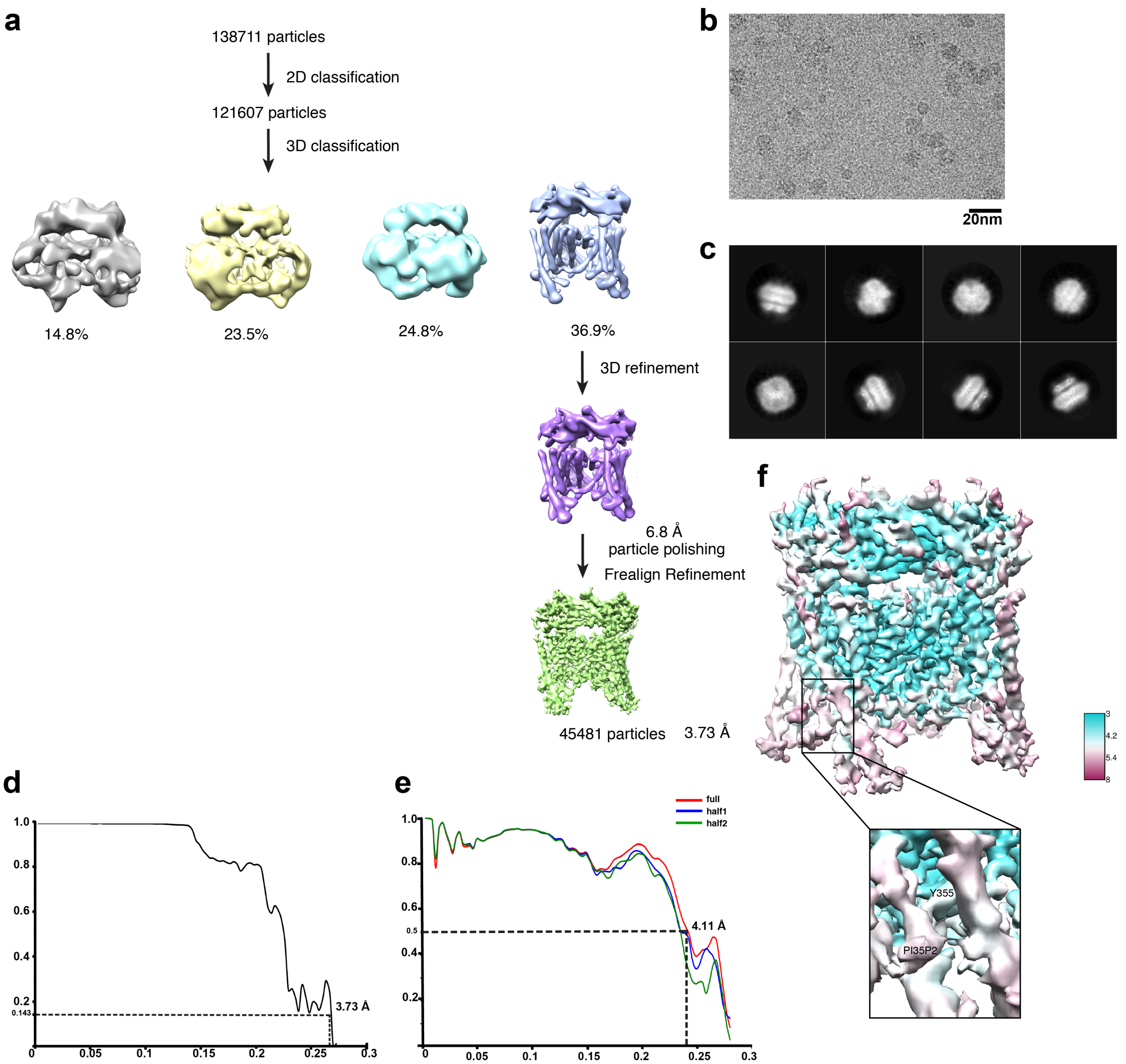

Supplementary Fig. 6 Data processing and model quality assessment of PtdIns(3,5)P_2/ML-SA1 bound TRPML1.

a, The data processing work-flow for PtdIns(3,5)P_2/ML-SA1 bound TRPML1. **b**, A representative electron micrograph at defocus -2.0 µm. **c,** 2D classification. **d,** Fourier shell correlation (FSC) curve of the structure with FSC as a function of resolution using Frealign output. **e,** The FSC curves calculated between the refined structure and the half map used for refinement, the other half map, and the full map. **f**, Density map of PtdIns(3,5)P_2/ML-SA1 bound TRPML1 structure colored by local resolution estimation using blocres.

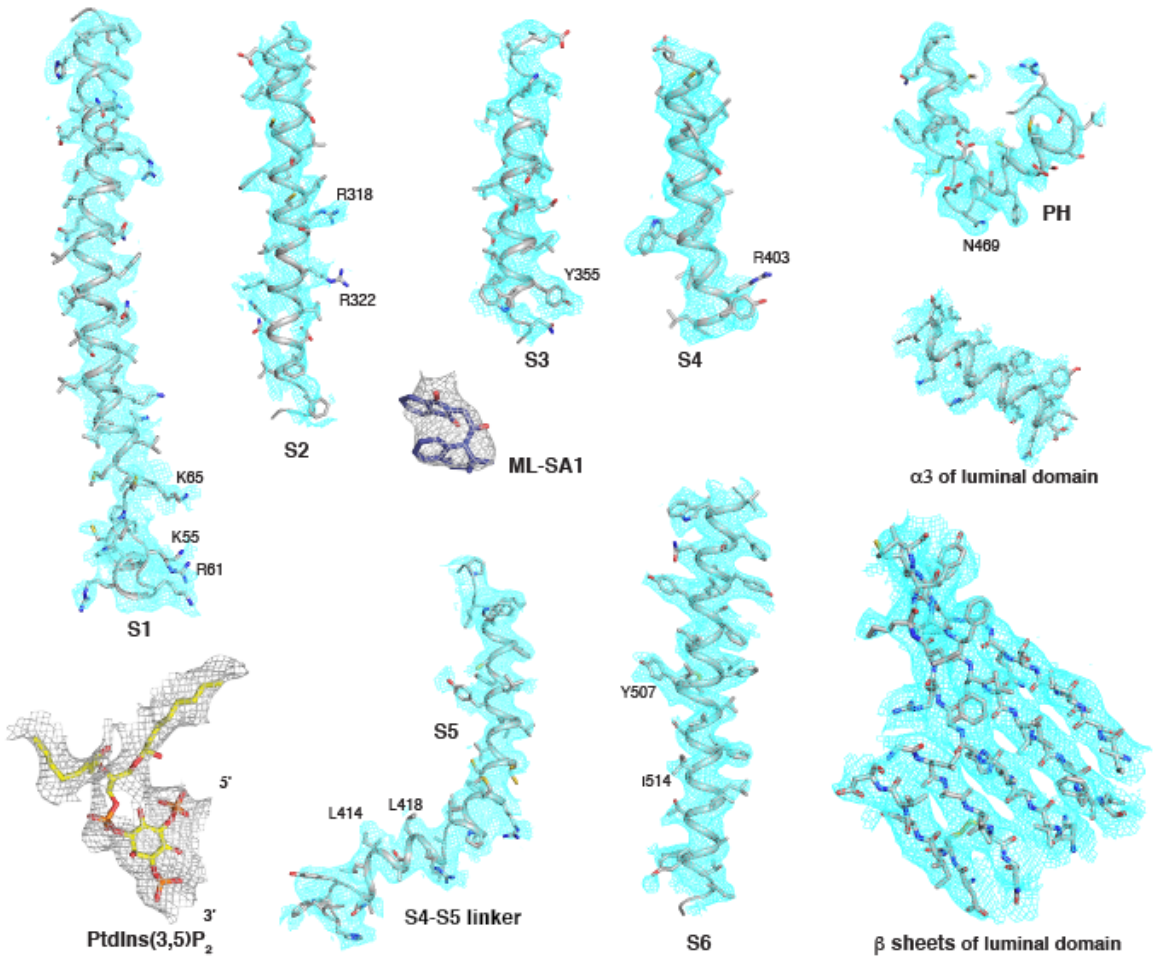

Supplementary Fig. 7 Electron microscopy density of different portions of PtdIns(3,5)P_2/ML-SA1 bound TRPML1 at 5σ level.

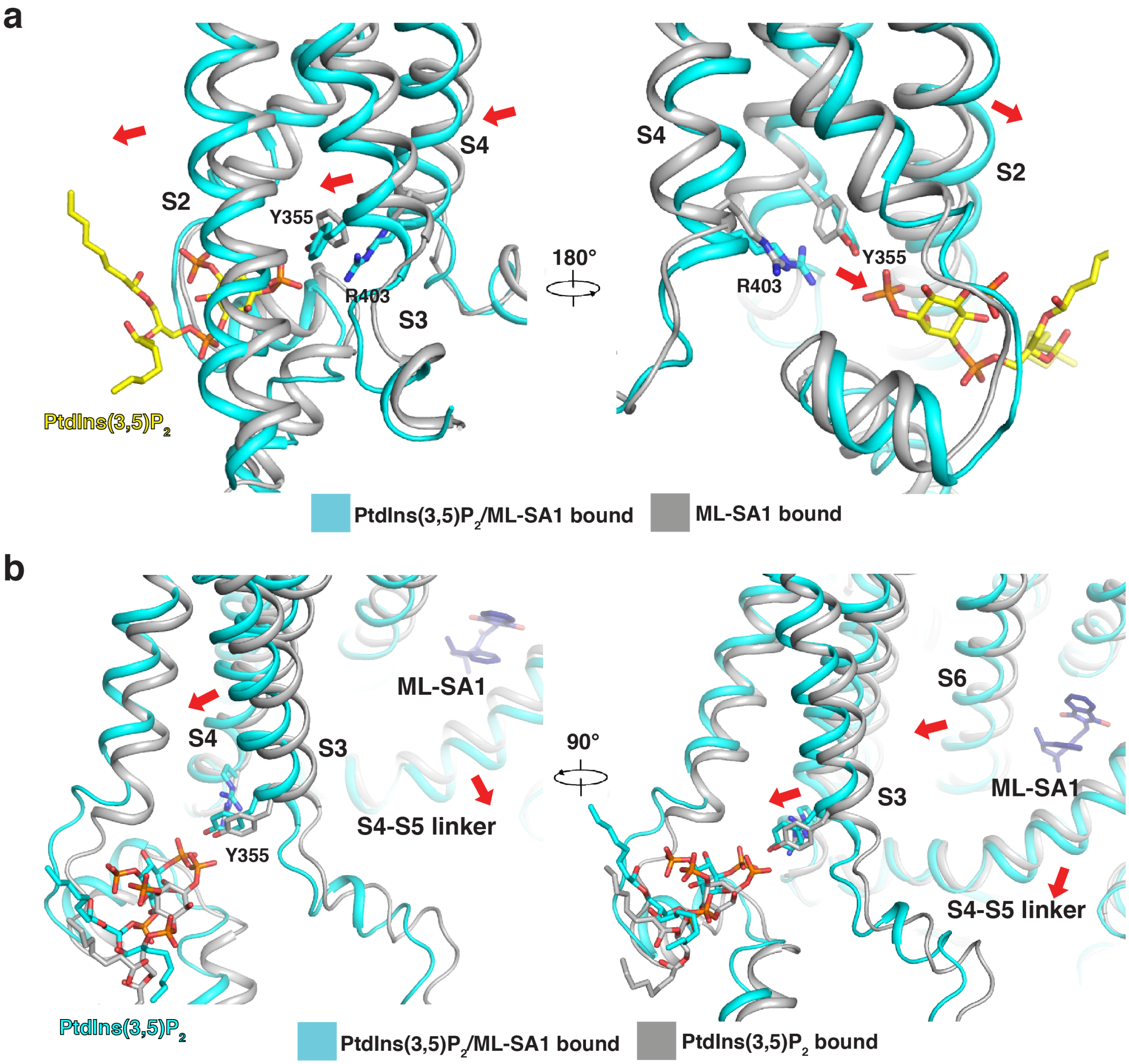

Supplementary Fig. 8 Structural comparison of PtdIns(3,5)P_2/ML-SA1 bound with ML-SA1 bound TRPML1 or PtdIns(3,5)P_2 bound TRPML1.

a, PtdIns(3,5)P_2/ML-SA1 bound TRPML1 is shown in cyan, with PtdIns(3,5)P_2 in yellow sticks. ML-SA1 bound TRPML1 is in gray with structural shifts indicated by red arrows.

b, PtdIns(3,5)P_2/ML-SA1 bound TRPML1 is shown in cyan, with PtdIns(3,5)P_2 in cyan sticks. PtdIns(3,5)P_2 bound TRPML1 is in gray, with PtdIns(3,5)P_2 in gray sticks. The structural shifts indicated by red arrows.

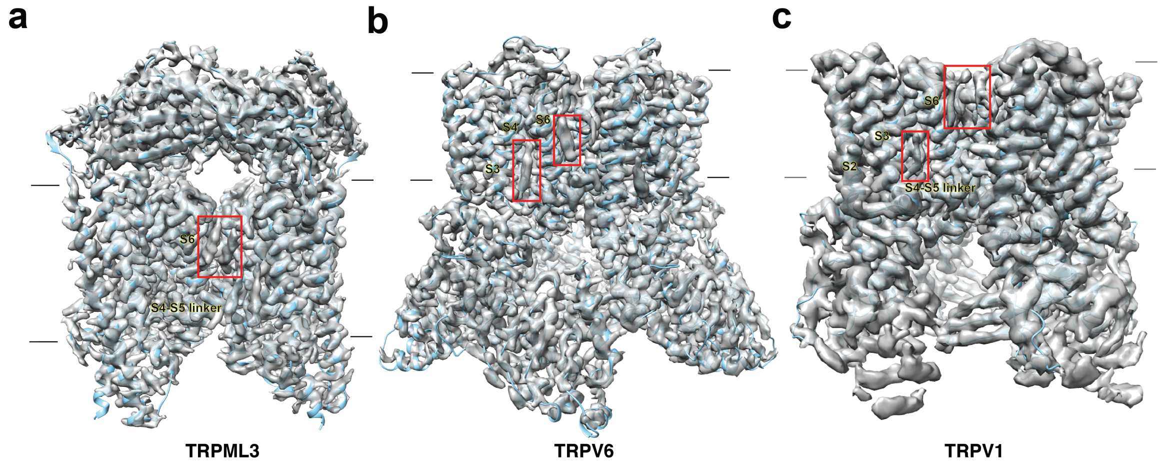

Supplementary Fig. 9 Lipid-bound sites of TRP channels.
a, cryo-EM map and structure of TRPML3 (EMD-8764 and PDB:5W3S). **b,** cryo-EM map and structure of TRPV6 (EMD-7120 and PDB:6BO8). **c,** cryo-EM map and structure of TRPV1 (EMD-8118 and PDB:5IRZ). The lipid-bound sites are indicated by red frames and the related structural elements are labeled.

Supplementary Table. 1 Cryo-EM data collection, refinement and validation statistics

	PI(3,5)P2-bound (EMDB-9000) (PDB 6E7P)	PI(4,5)P2-bound (EMDB-9001) (PDB 6E7Y)	PI(3,5)P2/ML-SA1-bound (EMDB-9002) (PDB 6E7Z)
**Data collection and processing**			
Magnification	46730	46730	46730
Voltage (kV)	300	300	300
Electron exposure (e–/Å^2)	84	84	84
Defocus range (μm)	-1.2 to -2.5	-0.8 to -2.3	-0.8 to -2.7
Pixel size (Å)	1.07	1.07	1.07
Symmetry imposed	C4	C4	C4
Initial particle images (no.)	108405	183600	138711
Final particle images (no.)	53482	69587	45481
Map resolution (Å)	3.51	3.57	3.73
FSC threshold	0.143	0.143	0.143
Map resolution range (Å)	3.51-256	3.57-256	3.73-256
**Refinement**			
Initial model used (PDB code)	5WJ5	5WJ5	5WJ5
Model resolution (Å)	3.51	3.57	3.73
FSC threshold	0.143	0.143	0.143
Model resolution range (Å)	3.51-256	3.57-256	3.73-256
Map sharpening *B* factor (Å^2)	-100	-100	-100
Model composition			
Non-hydrogen atoms	15508	15508	15616
Protein residues	1900	1900	1900
Ligands	4	4	8
B factors (Å^2)			
Protein	102.42	51.37	106.27
Ligand	143.62	109.59	124.88
R.m.s. deviations			
Bond lengths (Å)	0.0110	0.0044	0.0103
Bond angles (°)	1.29	1.14	1.26
Validation			
MolProbity score	3.62	2.1	4.96
Clashscore	1.65	1.43	1.83
Poor rotamers (%)	0.24	0	0
Ramachandran plot			
Favored (%)	91.8	92.9	89.2
Allowed (%)	8.0	7.1	10.8
Disallowed (%)	0.2	0	0

Supplementary Table. 2 Primer sequences

Primer Name	Primer Sequence
Y355A 5’	TTT GTC AAT GGC TGG GCC ATC CTG CTC GTC ACC
Y355A 3’	GGT GAC GAG CAG GAT GGC CCA GCC ATT GAC AAA
R403A 5’	GTG TGG GTG GGC GTG ATC GCA TAC CTG ACC TTC TTC CAC
R403A 3’	GTG GAA GAA GGT CAG GTA TGC GAT CAC GCC CAC CCA CAC
R403K 5’	GTG TGG GTG GGC GTG ATC AAA TAC CTG ACC TTC TTC CAC
R403K 3’	GTG GAA GAA GGT CAG GTA TTT GAT CAC GCC CAC CCA CAC
